# Real-life multidisciplinary treatment for unresectable colorectal cancer liver metastases including hepatic artery infusion with chemo-filtration and liquid biopsy precision oncotherapy: observational cohort study

**DOI:** 10.1007/s00432-020-03156-3

**Published:** 2020-02-22

**Authors:** Stefano Guadagni, Marco Clementi, Andrew R. Mackay, Enrico Ricevuto, Giammaria Fiorentini, Donatella Sarti, Paola Palumbo, Panagiotis Apostolou, Ioannis Papasotiriou, Francesco Masedu, Marco Valenti, Aldo Victor Giordano, Gemma Bruera

**Affiliations:** 1 Department of Applied Clinical and Biotechnological Sciences, University of L’Aquila, 67100 L’Aquila, Italy; 2 Oncology Territorial Care S. Salvatore Hospital, Oncology Network ASL1 Abruzzo, L’Aquila, Italy; 3 Department of Oncology and Hematology, Azienda Ospedaliera “Ospedali Riuniti Marche Nord”, Pesaro, Italy; 4 Department of Life, Health and Environmental Sciences, University of L’Aquila, 67100 L’Aquila, Italy; 5 Research Genetic Cancer Centre S.A, Florina, Greece; 6 Research Genetic Cancer Centre International GmbH, Zug, Switzerland; 7 Interventional Radiology, S. Salvatore Hospital, 67100 L’Aquila, Italy

**Keywords:** Liver metastases, Colorectal cancer, Third-line therapy, Hepatic artery infusion, Liquid biopsies, Precision oncotherapy, Chemo-filtration

## Abstract

**Background:**

Hepatic artery infusion (HAI) and drug selection by liquid biopsy precision oncotherapy are under investigation for the multidisciplinary treatment of unresectable colorectal liver metastases (CRCLM) in progression after systemic therapy. Here, we compare the safety and efficacy of third-line HAI followed by target therapy with drug regimes selected by liquid biopsy precision oncotherapy to third-line systemic therapy with drug regimes selected partly by tissue biopsy precision oncotherapy, in a retrospective real-life study of 106 unresectable CRCLM patients.

**Methods:**

Drug regimens for HAI/target therapy were selected by assessing the sensitivity of purified circulating tumor cell (CTCs) to 5-fluorouracil, carboplatin, cisplatin, oxaliplatin, irinotecan, doxorubicin, mitomycin, raltitrexed, and melphalan in-vitro and by real-time qRT-PCR gene expression assays, and for the Systemic therapy cohort were selected by age, comorbidity, performance status, and absence of RAS mutations. Therapeutic responses, adverse events, and quality of life were evaluated by RECIST 1.1, CTCAE 4.03, and ECOG criteria, respectively, and chemo-filtration performed following HAI to reduce systemic toxic effects.

**Results:**

HAI/target therapy with drugs selected by liquid biopsy precision oncotherapy (44 patients), resulted in 2.27% CRs, 38.63% PRs, 56.81% SD,s and 2.27% PDs; ECOG 2 to 1 improvement, but no infusion-related technical or vascular complications, or deaths. Systemic therapy (62 patients) resulted in 1.6% CRs, 17.74% PRs, 37.09% SDs, and 45.16% PDs; more grade 1–2 adverse events and 4.84% ECOG 1 to 2 worsening. The median 5 month PFS in the HAI/target therapy cohort was significantly longer than 3 months in the systemic cohort (*P* < 0.007) and the median 14 month survival in the HAI/target therapy cohort was longer than 8.5 months in the systemic therapy cohort but not statistically significant. Multivariate analysis identified ECOG grade 2 as the most unfavourable survival prognostic factor in both cohorts.

**Conclusions:**

HAI plus chemo-filtration followed by target therapy, with drug regimens selected by liquid biopsy precision oncotherapy, is a safe and efficacious alternative therapeutic strategy for unresectable CRCLM in progression after two lines of systemic therapy and should be considered for a multicentre prospective phase III study, to fully confirm this potential.

## Introduction

Thirty-to-sixty percent of colorectal cancer patients develop liver metastases (Hugen et al. 2014), of which only 15–20% are resectable at presentation (Datta et al. 2019). Neoadjuvant chemotherapy and improvements in surgical resection criteria have increased survival rates at 5 and 10 years by 40% and 25%, respectively (Chapelle et al. 2018). However, post-resection 1 year and overall relapse rates of 30% at 60%, respectively, have been reported (Chapelle et al. 2018).

Over the past decade, the first and second-line systemic therapy regimes for patients with unresectable and recurrent colorectal cancer liver metastases (CRCLM) with wild-type RAS have included 5-fluorouracil, irinotecan, oxaliplatin, and capecitabine combined with cetuximab, with bevacizumab preferred for RAS-mutated tumours (Fiorentini et al. 2017a).

Loco-regional chemoembolization (TACE) (Fiorentini et al. 2017b,^2018^; Guo et al. 2017; Wei et al. 2019) and hepatic artery infusion (HAI) are considered for the third-line therapy in unresectable CRCLM, with the latter indicated for elderly patients with poor performance status and those who refuse surgery or systemic chemotherapy or progress following systemic chemotherapy or to prolong intervals between cycles of systemic chemotherapy (Fiorentini et al. 2017a). Risks associated with HAI include treatable catheter and/or port/pump placement complications, life-threatening biliary sclerosis, hepatotoxicity, and/or systemic toxicity (Kingham et al. 2010), which can be reduced by chemo-filtration (Fiorentini et al. 2004a).

HAI has also been investigated as first-line (Fiorentini et al. 2004a; D’Angelica et al. 2015; Lim et al. 2017) and second-line therapy, with drug regimens including 5-fluorodeoxyuridine (FUDR) (Fiorentini et al. 2006,2005), pirarubicin (Fallik et al. 2003), irinotecan (Fiorentini et al. 2003), oxaliplatin (Fiorentini et al. 2004b), and, more recently, intraarterial oxaliplatin/irinotecan/5-fluorouracil followed by intravenous cetuximab (OPTILIV trial) (Lévi et al. 2016).

We previously reported that the first-line HAI therapy for unresectable CRCLM results in median overall survival times of 17 months for mitomycin (30 mg/m^2^) and epirubicin (60 mg/m^2^) (Fiorentini et al. 2004a), 20 months for FUDR (0.25 mg/kg/day for 14 days) with intravenous 5-fluorouracil (400 mg/m^2^ for 4 days) (Fiorentini et al. 2006) and, as second-line therapy, 13 months for both irinotecan (200 mg/m^2^) (Fiorentini et al. 2003) and oxaliplatin (150 mg/m^2^) (Fiorentini et al. 2004b).

Considering that tissue biopsy precision oncotherapy and chemosensitivity tests (Yoon and Kim 2014) have been recommended for further investigation (Sepulveda et al. 2017), liquid biopsy has been validated and approved as prognostic method (Karachaliou et al. 2015; Goodsaid 2019) and our previous results, we hypothesized that the third-line HAI therapy with drug regimens selected by liquid biopsy precision oncotherapy could provide a better local disease control and improve overall survival in patients with unresectable CRCLM.

In accordance with this, we report a real-life retrospective cohort study of unresectable CRCLM in progression after two lines of therapy, in which we evaluate and compare the safety and efficacy of third-line HAI with drug regimens selected by liquid biopsy precision oncotherapy followed by target therapy, to third and subsequent line systemic therapy regimens, selected in part by tissue biopsy precision oncotherapy.

## Materials and methods

This study involved patients with unresectable and predictable disease course, was approved by ASL n.1 Ethics committee, Abruzzo, Italy (Chairperson: G. Piccioli; protocol number 10/CE/2018; approved: 19 July 2018 (n.1419)] and undertaken at the University of L'Aquila, L'Aquila, Italy. All patients provided written consent and received complete information about their disease and the implications of the proposed treatment, in accordance with the Helsinki Declaration and the University of L’Aquila committee on human experimentation.

### Patient cohorts

In 106 patients diagnosed with unresectable CRCLM in progression after two lines of systemic chemotherapy between 2007 and 2018, 44 received HAI plus chemo-filtration, with drug regimens selected by liquid biopsy precision oncotherapy followed by target therapy and 62 received systemic therapy with drug regimens selected in part by tissue biopsy precision oncotherapy. Decisions concerning un-resectability and precision oncotherapy were made by experienced hepato-biliary surgeons, oncologists, and radiologists during multidisciplinary meetings. Inclusion criteria were: (1) histologically confirmed colorectal cancer diagnosis and complete primary tumor resection; (2) unresectable liver metastases and contraindications for liver resection; (3) failure of two lines of systemic chemotherapy; (4) Eastern Cooperative Oncology Group (ECOG) performance status of < 3; (5) tumor involvement < 70% of liver volume with adequate liver or renal dysfunction (total bilirubin serum levels < 3 mg/dL, serum albumin level > 20 g/L, and serum creatinine level < 2 mg/dL). In all cases, systemic chemotherapy for liver metastases ceased 4 weeks prior to the first cycle of third-line therapy. Patients with extra-hepatic metastases were also included if the main lesions were in the liver. Patients with inadequate medical records were excluded from this study. Patient characteristics are reported in Table 1.

**Table 1:** Characteristics of 44 patients with unresectable CRCLM submitted to a multidisciplinary treatment including third-line HAI followed by target therapy based on liquid biopsy precision oncotherapy compared to 62 patients submitted to third-line systemic therapy

	All patients (*n* = 106);	HAI/target therapy cohort (*N* = 44)		Systemic therapy cohort (*N* = 62)		*P* value
Age (years)	Median (iqr)	Median	iqr	Median	iqr	n.s
	64 (59–70)	66.5	62.5–71.5	62	58–69	
Gender	*N* (%)	*N*	%	*N*	%	n.s
Female	42 (39.62)	19	43.18	23	37.10	
Male	64 (60.38)	25	56.82	39	62.90	
Primary tumor site		*N*	%	*N*	%	ns
Right hemicolon	27 (25.47)	10	22.73	17	27.42	
Left hemicolon	53 (50.00)	27	61.36	26	41.94	
Rectum	26 (24.53)	7	15.91	19	30.65	
Primary tumor pathological grade		*N*	%	*N*	%	n.s
Well or intermediate	54 (50.95)	23	52.27	31	50.00	
Poor	52 (49.05)	21	47.73	31	50.00	
Time to liver metastases		*N*	%	*N*	%	n.s
Synchronous	75 (70.75)	27	61.36	48	77.42	
Metachronous	31 (29.25)	17	38.64	14	22.58	
Bilateral liver involvement		*N*	%	*N*	%	n.s
Yes	64 (60.38)	22	50.00	42	67.64	
Not	42 (39.62)	22	50.00	20	32.26	
At least one metastasis with diameter > 5 cm		*N*	%	*N*	%	n.s
Yes	50 (47.17)	19	43.18	31	50.00	
Not	56 (52.83	25	56.82	31	50.00	
Extra-hepatic metastasis		*N*	%	*N*	%	n.s
Absence	41 (38.68)	22	50.00	19	30.65	
Presence	65 (61.32)	22	50.00	43	69.35	
Previous systemic chemotherapy for liver metastases		*N*	%	*N*	%	
First line	106	44	100	62	100	
5FU/OX/Leucovorin		24	54.55	6	9.68	0.001
5FU/IRI/Bevacizumab/OX		6	13.64	23	37.10	0.01
5FU		3	6.82	1	1.61	n.s
5FU/IRI/Leucovorin		6	13.64	0	0	0.04
5FU/IRI/Bevacizumab		1	2.27	5	8.06	n.s
5FU/OX/Bevacizumab		1	2.27	5	8.06	n.s
5FU/OX/Cetuximab		1	2.27	1	1.61	n.s
5FU/OX/IRI/leucovorin		2	4.55	3	4.84	n.s
Capecitabine		0	0	1	1.61	n.s
5FU/OX/IRI/cetuximab		0	0	10	16.13	0.005
OX/IRI		0	0	1	1.61	n.s
OX		0	0	2	3.22	n.s
OX/IRI/cetuximab		0	0	4	6.45	n.s
IRI/cetuximab		0	0	1	1.61	n.s
Second line	106	44	100	62	100	
Capecitabine		20	45.45	1	1.61	0.001
5FU/IRI		15	34.09	4	6.45	0.001
Capecitabine/OX		3	6.82	1	1.61	n.s
Capecitabine/Bevacizumab		1	2.27	3	4.84	n.s
5FU		1	2.27	1	1.61	n.s
5FU/IRI/Cetuximab		1	2.27	6	9.68	n.s
5FU/IRI/Panitumumab		1	2.27	0	0	n.s
OX/IRI/Cetuximab		1	2.27	2	3.22	n.s
5FU/Bevacizumab		0	0	2	3.22	n.s
IRI/Cetuximab		0	0	1	1.61	n.s
Raltitrexed		0	0	3	4.84	n.s
5FU/OX/ Panitumumab		0	0	1	1.61	n.s
5FU/IRI/Aflibercept		0	0	5	8.06	n.s
OX/IRI/Bevacizumab		0	0	3	4.84	n.s
IRI		0	0	2	3.22	n.s
OX/IRI		1	2.27	1	1.61	n.s
Capecitabine/IRI/Bevacizumab		0	0	1	1.61	n.s
5FU/IRI/Bevacizumab		0	0	3	4.84	n.s
5FU/OX/Bevacizumab		0	0	3	4.84	n.s
5FU/OX/IRI/Bevacizumab		0	0	10	16.13	0.005
5FU/OX/IRI/Cetuximab		0	0	6	9.68	n.s
OX/Cetuximab		0	0	1	1.61	n.s
IRI/Bevacizumab		0	0	3	4.84	n.s
Eastern Cooperative Oncology Group (ECOG)		*N*	%	*N*	%	0.001
2	29 (27.36)	20	45.45	9	14.52	
1	43 (40.57	24	54.55	19	30.65	
0	34 (32.07)	0	0	34	54.84	
KRAS, NRAS, BRAF genotype status	*n* = 106 (%)	*N*	%	*N*	%	n.s
No mutations	68 (64.15)	30	68.18	38	61.29	
Mutations	38 (35.85)	14	31.82	24	38.71	
KRAS exon 2 codon 12		− 11	– (78.6%)	18	– (75%)	
KRAS exon 2 codon 13		− 0	– (0%)	3	– (12.5%)	
KRAS exon 3 codon 61		− 0	– (0%)	1	– (4.17%)	
NRAS exon 3 codon 61		− 2	– (14.3%)	0	– (0%)	
NRAS exon 2 codon 12		− 0	– (0%)	3	– (12.5%)	

*SD* standard deviation, *iqr* interquartile range, *n.s.* not significant, *5FU* 5-fluorouracil, *IRI* irinotecan, *OX* oxaliplatin

### HAI with chemo-filtration

Following local anesthesia, femoral artery puncture was performed using the Seldinger technique. Size, number, position, and blood supply of liver lesions were determined by introduction of 5F-RH catheters (Boston Scientific, USA) into the abdominal aorta, celiac trunk, and superior mesenteric artery, by digital subtraction angiography (DSA), which also detected aberrant and accessory hepatic arteries. A 3F microcatheter (Terumo Corp, Tokyo, Japan) was then used for super-selective intubation of the hepatic artery. Aberrant or accessory hepatic arteries were also used for drug infusion, if found to be the main tumor-nourishing vessels. Drugs were perfused at infusion rate of 14–20 mL/min, and antiemetics and antacids were routinely used for hepatoprotection during HAI.

The 5F arterial and venous introducers, the latter positioned in a femoral or humeral vein, were connected to a hypoxic perfusion tubing-set containing a polyamide haemofilter of 2.1 m^2^ surface area (RanD, Medolla, Italy) mounted on a dedicated extracorporeal circulation and chemo-filtration device (Performer LRT; RanD, Medolla, Italy), containing a heating element to avoid hypothermia, and the circuit primed with heparin-containing isotonic sodium chloride solution (10,000 U/L). Chemo-filtration (≈ 40 min) was initiated upon establishing a blood flow of ≈ 100 ml/min in the extracorporeal circuit (aspiration from the arterial and infusion into venous introducers) and was followed by introducer withdrawal, vessel compression for ≈30 min, and injection of protamine (200 IU/kg) to reverse heparin-dependent coagulation inhibition.

### Liquid biopsy precision, chemosensitivity, and tumor gene expression assays

Liquid biopsy precision oncotherapy, chemosensitivity, and tumor cell gene expression assays have all been described previously (Guadagni et al. 2020; Apostolou et al. 2013, 2017,2019). Briefly, for each patient, 20 mL of blood were collected in sterile 50 ml Falcon tubes (4440100, Orange Scientific, Braine-l’Alleud, Belgium), containing 7 ml of 0.02 M EDTA anticoagulant (E0511.0250, Duchefa Biochemie B.V., Haarlem, The Netherlands), stored at 2–8 °C in impact-resistant transportation containers to ensure the stability and viability of circulating tumor cells (CTCs), and analyzed within 80 h for the presence of > 5 viable circulating tumor cells (CTCs) per ml (Apostolou et al. 2017). For CTCs purification, blood samples were layered over 4 ml polysucrose solution (Biocoll separating solution 1077, Biochrom, Berlin, Germany) and centrifuged for 20 min at 2500×*g*. Mononuclear cells, lymphocytes, platelets, and granulocytes were collected and washed with phosphate-buffered saline (PBS, P3813; Sigma-Aldrich, Germany), incubated for 10 min in lysis buffer (154 mM NH_4_Cl (31,107; Sigma-Aldrich), 10 mM KHCO_3_ (4854; Merck, Germany) and 0.1 mM EDTA in deionized water) to lyse erythrocytes, centrifuged, re-washed in PBS then incubated with CD45-conjugated magnetic beads (39-CD45-250; Gentaur, Belgium) and pan-cytokeratin (pan-CK)-conjugated microbeads (MA1081-M; Gentaur) for 30 min at 4 °C. Following incubation, cells were collected in a magnetic field, washed in PBS, and purified pan-cytokeratin positive/CD45-negative cells cultured in 12-well plates (4430400 N; Orange Scientific) in RPMI-1640 plus 10% FBS for chemosensitivity, viability, and qRT-PCR assays. Purified peripheral blood mononuclear cells (PBMCs) from each patient were used as non-cancer cell controls. CTCs were validated by qRT-PCR, using specific primers for CK19 and pan-CK, and other cell types excluded using primers for CD31 and N-cadherin. Samples for chemosensitivity and gene expression assays contained ≥ 5 viable circulating tumor cells/ml.

Chemosensitivity assays were performed as previously described for colorectal cancer patients (Apostolou et al. 2013). Briefly, cells cultured in 12-well plates (3513, Corning) were treated with the following drug concentrations: 1 μM melphalan (Μ2011, Sigma-Aldrich), 1 μM doxorubicin (D1515, Sigma-Aldrich), 1 μM cisplatin (P4394, Sigma-Aldrich), 10 μM 5-fluorouracil (F6627, Sigma-Aldrich), 1.12 μM oxaliplatin (O9512, Sigma-Aldrich), 1 μM carboplatin (41575-94-4, Sigma-Aldrich), 5 μM irinotecan (I1406, Sigma-Aldrich), 1 μM raltitrexed (112887-68-0, Sigma-Aldrich), and 2 μM mitomycin C (M4287, Sigma-Aldrich), and cell viability was assessed by flow cytometry (BD Instruments Inc., San José, CA) at 24-h intervals for 6 days, using Annexin V-PE (559763; BD Bioscience), and the percentage of living, dead and apoptotic cells evaluated, using BD CellQuest Software (BD Instruments Inc). Chemosensitivity and viability validation were also corroborated by methyl-tetrazolium dye (MTT), crystal violet dye (CVE), and Sulfo-Rodhamine B (SRB) assays. The percentage of non-viable cancer cells was calculated under non-drug and drug-treated conditions, and chemosensitivity classified as: (1) non-sensitive < 35%; (2) partially sensitive 35–80%, and (3) highly sensitive > 80%.

For gene expression, RNAs was purified from CRCLM patient CTCs, using RNeasy Mini Kits, as directed (74105, Qiagen, Hilden, Germany), 1 µg of RNA reverse transcribed using a PrimeScript RT Reagent Kit, as directed (RR037A, Takara, Beijing, China) and subjected to KAPA SYBR Fast Master Mix (2 ×) Universal (KK4618, KAPA Biosystems, MA, USA) real-time qPCR (Apostolou et al. 2013,2019) in a final volume of 20 μl, using specific primers and appropriate housekeeping genes (Apostolou et al. 2019). In PCR reactions, denaturation at 95 °C for 2 min was followed by 40 PCR cycles consisting of 10 s denaturation at 95 °C, 30 s annealing at 59 °C. Melting-curve analysis was performed from 70–90 °C, with 0.5 °C increments of 5 s, at each step. All reactions were performed in triplicate, compared to template-free negative controls and analyzed by Livak relative quantification (Apostolou et al. 2017). Gene expression was compared in patient CTCs pre- and post-treatment and quantified using the following equations:

$$\Delta Ct_{{({\text{threshold Cycle}})}} = \, Ct_{{{\text{target}}}} - \, Ct_{{{\text{18SrRNA}}}}$$

$$\Delta \Delta Ct \, = \, \Delta Ct_{{({\text{treated CTCs}})}} - \, \Delta Ct_{{({\text{non}} - {\text{cancer cells}})}}$$

$${\text{Relative expression level }} = { 2}^{ - \Delta \Delta Ct}$$

$$\% {\text{ Gene expression }} = { 1}00 \, \times \, \left( {{2}^{ - \Delta \Delta Ct} - {1}} \right),$$

and classified as either: low over-expression (< 50%) or high over-expression (> 50%). A more detailed description of the gene expression panel for colorectal cancer patients is reported elsewhere (Apostolou et al. 2019).

### Drug regimens

**Fig. 1:**
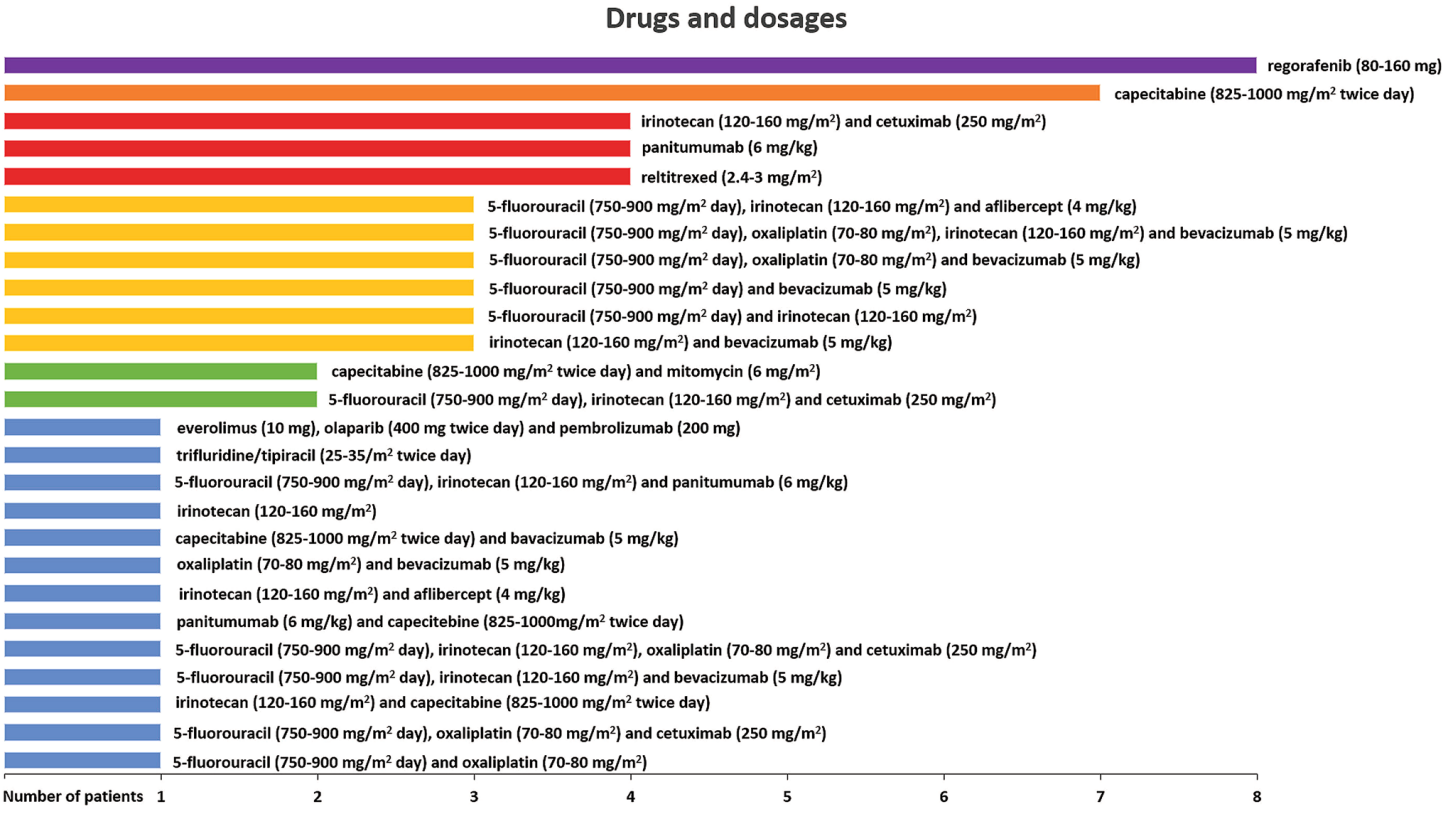
Third-line systemic therapeutic regimens selected for the 62 patient cohort with unresectable CRCLM in progression after two lines of systemic therapy

Drug regimens in the HAI/target-therapy cohort (44 patients) were selected according to the published criteria (Guadagni et al. 2020) and are reported in Table 2. Irinotecan, 5-fluorouracil, oxaliplatin, and mitomycin were chosen based on chemosensitivity assays, previous systemic chemotherapy protocols, drug allergies, or intolerance and dosage based on previous pharmacokinetic, dose finding, and clinical studies (Fiorentini et al. 2004b, 2005; Kern et al. 2001; Guadagni et al. 2000). In the systemic therapy cohort (62 patients), systemic therapeutic regimens reflected the clinical parameters of age, comorbidity, performance status, and absence of KRAS and NRAS mutations in exon 2 (codons 12 and 13), exon 3 (codons 59 and 61) and/or exon 4 (codons 117 and 146) in recurrent cancer cells or primary tumor biopsies, as reported in Fig. 1 and previously described (Guadagni et al. 2019; Bruera et al. 2018).

**Table 2:** Liquid biopsy chemosensitivity (Part-A) and tumor gene expression (Part-B) assays in the HAI/target-therapy cohort

Part-A																		
Pt		IV-CTCs		5-FU	Oxaliplatin	Irinotecan		Mitomycin	Doxorubicin	Cisplatin	Alkeran		Carboplatin		Raltitrexed		TC based on CST	
1		9.8/ml		*S* = 58%	*S* = 62%	*S* = 80%		*S* = 86%	*S* = 20%	*S* = 70%	*S* = 25%		*S* = 45%		*S* = 20%		MMC (30 mg/m^2^)	
2		16.8/ml		*S* = 81%	*S* = 80% (A)	*S* = 20%		*S* = 82%	*S* = 26%	*S* = 81%	*S* = 25%		*S* = 75%		*S* = 60%		MMC (30 mg/m^2^)	
3		14.8/ml		*S* = 70%	*S* = 75%	*S* = 70%		*S* = 82%	*S* = 19%	*S* = 80%	*S* = 25%		*S* = 70%		*S* = 60%		MMC (30 mg/m^2^)	
4		8.9/ml		*S* = 65%	*S* = 70%	*S* = 82%		*S* = 82%	*S* = 24%	*S* = 65%	*S* = 24%		*S* = 80%		*S* = 70%		MMC (30 mg/m^2^), IRI (200 mg/m^2^)	
5		15.3/ml		*S* = 65%	*S* = 70%	*S* = 60%		*S* = 81%	*S* = 22%	*S* = 65%	*S* = 23%		*S* = 60%		*S* = 45%		MMC (30 mg/m^2^), OX (150 mg/m^2^)	
6		9.8/ml		*S* = 25%	*S* = 45%	*S* = 75%		*S* = 85%	*S* = 24%	*S* = 40%	*S* = 20%		*S* = 42%		*S* = 20%		MMC (30 mg/m^2^), IRI (200 mg/m^2^)	
7		8.9/ml		*S* = 70%	*S* = 80%	*S* = 55%		*S* = 82%	*S* = 22%	*S* = 75%	*S* = 24%		*S* = 65%		*S* = 70%		MMC (30 mg/m^2^)	
8		12.2/ml		*S* = 48%	*S* = 61%	*S* = 48%		*S* = 82%	*S* = 23%	*S* = 70%	*S* = 23%		*S* = 47%		*S* = 28%		MMC (30 mg/m^2^)	
9		16.2/ml		*S* = 60%	*S* = 72%	*S* = 42%		*S* = 45%	*S* = 20%	*S* = 62%	*S* = 56%		*S* = 57%		*S* = 60%		5-FU (600 mg/m^2^), OX (150 mg/m^2^)	
10		9.8/ml		*S* = 70%	*S* = 70%	*S* = 70%		*S* = 46%	*S* = 25%	*S* = 65%	*S* = 24%		*S* = 55%		*S* = 25%		5-FU (600 mg/m^2^), OX (150 mg/m^2^)	
11		9.4/ml		*S* = 25%	*S* = 55%	*S* = 75%		*S* = 60%	*S* = 30%	*S* = 50%	*S* = 35%		*S* = 45%		*S* = 60%		MMC (30 mg/m^2^), IRI (200 mg/m^2^)	
12		7.5/ml		*S* = 62%	*S* = 75%	*S* = 85%		*S* = 58%	*S* = 22%	*S* = 55%	*S* = 20%		*S* = 70%		*S* = 38%		OX (150 mg/m^2^), IRI (200 mg/m^2^)	
13		8.9/ml		*S* = 70%	*S* = 80%	*S* = 85%		*S* = 25%	*S* = 24%	*S* = 70%	*S* = 40%		*S* = 60%		*S* = 25%		5-FU (600 mg/m^2^), OX (150 mg/m^2^), IRI (200 mg/m^2^)	
14		9.3/ml		*S* = 35%	*S* = 50%	*S* = 80%		*S* = 30%	*S* = 35%	*S* = 45%	*S* = 30%		*S* = 54%		*S* = 50%		IRI (200 mg/m^2^)	
15		15.1/ml		*S* = 40%	*S* = 55%	*S* = 80%		*S* = 60%	*S* = 30%	*S* = 45%	*S* = 75%		*S* = 55%		*S* = 25%		IRI (200 mg/m^2^)	
16		16.3/ml		*S* = 50%	*S* = 60%	*S* = 80%		*S* = 25%	*S* = 20%	*S* = 45%	*S* = 75%		*S* = 60%		*S* = 10%		IRI (200 mg/m^2^)	
17		8.7/ml		*S* = 55%	*S* = 70%	*S* = 40%		*S* = 75%	*S* = 24%	*S* = 65%	*S* = 24%		*S* = 55%		*S* = 20%		MMC (30 mg/m^2^), OX (150 mg/m^2^)	
18		14.1/ml		*S* = 75%	*S* = 75%	*S* = 75%		*S* = 30%	*S* = 30%	*S* = 60%	*S* = 40%		*S* = 60%		*S* = 25%		5-FU (600 mg/m^2^), OX (150 mg/m^2^), IRI (200 mg/m^2^)	
19		8.8/ml		*S* = 50%	*S* = 70%	*S* = 25%		*S* = 75%	*S* = 35%	*S* = 65%	*S* = 20%		*S* = 55%		*S* = 28%		MMC (30 mg/m^2^), OX (150 mg/m^2^)	
20		12.2/ml		*S* = 50%	*S* = 70%	*S* = 30%		*S* = 75%	*S* = 24%	*S* = 60%	*S* = 24%		*S* = 55%		*S* = 25%		MMC (30 mg/m^2^), OX (150 mg/m^2^)	
21		9.8/ml		*S* = 48%	*S* = 60%	*S* = 85%		*S* = 80%	*S* = 60%	*S* = 45%	*S* = 50%		*S* = 35%		*S* = 28%		IRI (200 mg/m^2^)	
22		10/ml		*S* = 50%	*S* = 60%	*S* = 90%		*S* = 80%	*S* = 60%	*S* = 85%	*S* = 25%		*S* = 65%		*S* = 65%		IRI (200 mg/m^2^)	
23		7.5/ml		*S* = 75%	*S* = 80%	*S* = 25%		*S* = 25%	*S* = 25%	*S* = 55%	*S* = 40%		*S* = 60%		*S* = 50%		5-FU (600 mg/m^2^), OX (150 mg/m^2^)	
24		9.3/ml		*S* = 50%	*S* = 81%	*S* = 90%		*S* = 85%	*S* = 20%	*S* = 61%	*S* = 21%		*S* = 62%		*S* = 40%		IRI (200 mg/m^2^)	
25		16.2/ml		*S* = 60%	*S* = 65%	*S* = 80%		*S* = 65%	*S* = 72%	*S* = 60%	*S* = 20%		*S* = 48%		*S* = 65%		IRI (200 mg/m^2^)	
26		7.5/ml		*S* = 55%	*S* = 50%	*S* = 50%		*S* = 81%	*S* = 45%	*S* = 65%	*S* = 70%		*S* = 50%		*S* = 30%		MMC (30 mg/m^2^)	
27		12.2/ml		*S* = 25%	*S* = 70%	*S* = 60%		*S* = 70%	*S* = 55%	*S* = 70%	*S* = 40%		*S* = 55%		*S* = 65%		MMC (30 mg/m^2^), OX (150 mg/m^2^)	
28		15.1/ml		*S* = 45%	*S* = 85%	*S* = 30%		*S* = 90%	*S* = 31%	*S* = 35%	*S* = 44%		*S* = 45%		*S* = 75%		MMC (30 mg/m^2^), OX (150 mg/m^2^)	
29		7.5/ml		*S* = 50%	*S* = 55%	*S* = 80%		*S* = 70%	*S* = 20%	*S* = 60%	*S* = 70%		*S* = 62%		*S* = 27%		IRI (200 mg/m^2^)	
30		9.8/ml		*S* = 37%	*S* = 85	*S* = 42%		*S* = 65%	*S* = 65	*S* = 60%	*S* = 49%		*S* = 50%		*S* = 25%		OX (150 mg/m^2^)	
31		8.9/ml		*S* = 45%	*S* = 70%	*S* = 20%		*S* = 25%	*S* = 20%	*S* = 60%	*S* = 30%		*S* = 60%		*S* = 10%		OX (150 mg/m^2^)	
32		9/ml		*S* = 53%	*S* = 55%	*S* = 35%		*S* = 85%	*S* = 35%	*S* = 55%	*S* = 23%		*S* = 45%		*S* = 20%		MMC (30 mg/m^2^)	
33		15.1/ml		*S* = 55%	*S* = 83%	*S* = 70%		*S* = 20%	*S* = 25%	*S* = 60%	*S* = 20%		*S* = 62%		*S* = 50%		OX (150 mg/m^2^)	
34		8.3/ml		*S* = 55%	*S* = 60%	*S* = 45%		*S* = 80%	*S* = 24%	*S* = 50%	*S* = 40%		*S* = 50%		*S* = 20%		MMC (30 mg/m^2^)	
35		8.4/ml		*S* = 40%	*S* = 85%	*S* = 50%		*S* = 20%	*S* = 20%	*S* = 50%	*S* = 22%		*S* = 55%		*S* = 75%		OX (150 mg/m^2^)	
36		8.3/ml		*S* = 50%	*S* = 80%	*S* = 65%		*S* = 60%	*S* = 40%	*S* = 70%	*S* = 20%		*S* = 55%		*S* = 20%		OX (150 mg/m^2^)	
37		8.4/ml		*S* = 70%	*S* = 75%	*S* = 60%		*S* = 81%	*S* = 24%	*S* = 75%	*S* = 24%		*S* = 65%		*S* = 60%		MMC (30 mg/m^2^)	
38		15.2/ml		*S* = 60%	*S* = 60%	*S* = 40%		*S* = 85%	*S* = 20%	*S* = 60%	*S* = 45%		*S* = 50%		*S* = 20%		MMC (30 mg/m^2^)	
39		9.8/ml		*S* = 38%	*S* = 35%	*S* = 41%		*S* = 91%	*S* = 30%	*S* = 60%	*S* = 49%		*S* = 50%		*S* = 10%		MMC (30 mg/m^2^)	
40		6.9/ml		*S* = 55%	*S* = 65%	*S* = 40%		*S* = 85%	*S* = 24%	*S* = 55%	*S* = 24%		*S* = 60%		*S* = 25%		MMC (30 mg/m^2^)	
41		9.7/ml		*S* = 70%	*S* = 82%	*S* = 75%		*S* = 83%	*S* = 65%	*S* = 80%	*S* = 22%		*S* = 75%		*S* = 70%		MMC (30 mg/m^2^)	
42		6.9/ml		*S* = 35%	*S* = 42%	*S* = 50%		*S* = 80%	*S* = 35%	*S* = 55%	*S* = 65%		*S* = 48%		*S* = 40%		MMC (30 mg/m^2^)	
43		9.8/ml		*S* = 60%	*S* = 72%	*S* = 42%		*S* = 70%	*S* = 23%	*S* = 60%	*S* = 65%		*S* = 70%		*S* = 28%		MMC (30 mg/m^2^)	
44		9.9/ml		*S* = 50%	*S* = 70%	*S* = 40%		*S* = 75	*S* = 20%	*S* = 50%	*S* = 50%		*S* = 60%		S020%		MMC (30 mg/m^2^), OX (150 mg/m^2^)	

Part B																		
Pt	EGFR		VEGFR		*KRAS*	*NRAS*	*BRAF*	*MDR1*	*TYMS*	DHFR		SHMT1		ERCC1		GST		Target therapy
1	OE = 40%		OE = 45%		NM	NM	NM	OE = 80%	OE = 0%	OE = 0%		OE = 0%		OE = 0%		OE = 10%		
2	OE = 45%		OE = 45%		NM	NM	NM	OE = 65%	OE = 0%	OE = 25%		OE = 0%		OE = 0%		OE = 25%		
3	OE = 35%		OE = 45%		NM	NM	NM	OE = 65%	OE = 0%	OE = 0%		OE = 0%		OE = 15%		OE = 20%		
4	OE = 50%		OE = 50%		MT^12e2^	NM	NM	OE = 60%	OE = 10%	OE = 0%		OE = 0%		OE = 20%		OE = 20%		
5	OE = 35%		OE = 45%		MT^12e2^	NM	NM	OE = 55%	OE = 0%	OE = 0%		OE = 0%		OE = 25%		OE = 20%		
6	OE = 25%		OE = 40%		MT^12e2^	NM	NM	OE = 80%	OE = 10%	OE = 0%		OE = 0%		OE = 20%		OE = 20%		
7	OE = 50%		OE = 35%		NM	NM	NM	OE = 65%	OE = 5%	OE = 25%		OE = 10%		OE = 0%		OE = 15%		
8	OE = 65%		OE = 70%		NM	NM	NM	OE = 80%	OE = 0%	OE = 0%		OE = 0%		OE = 0%		OE = 18%		Bevacizumab (5 mg/kg)
9	OE = 55%		OE = 70%		NM	NM	NM	OE = 80%	OE = 0%	OE = 0%		OE = 0%		OE = 0%		OE = 10%		Bevacizumab (5 mg/kg)
10	OE = 35%		OE = 40%		NM	NM	NM	OE = 40%	OE = 0%	OE = 0%		OE = 0%		OE = 0%		OE = 5%		
11	OE = 35%		OE = 45%		NM	NM	NM	OE = 62%	OE = 0%	OE = 0%		OE = 0%		OE = 0%		OE = 10%		
12	OE = 60%		OE = 85%		NM	NM	NM	OE = 70%	OE = 0%	OE = 0%		OE = 0%		OE = 0%		OE = 10%		Bevacizumab (5 mg/kg)
13	OE = 36%		OE = 40%		NM	NM	NM	OE = 30%	OE = 0%	OE = 0%		OE = 0%		OE = 0%		OE = 0%		
14	OE = 20%		OE = 20%		NM	NM	NM	OE = 80%	OE = 10%	OE = 0%		OE = 0%		OE = 25%		OE = 20%		
15	OE = 50%		OE = 20%		MT^12e2^	NM	NM	OE = 60%	OE = 0%	OE = 0%		OE = 0%		OE = 0%		OE = 10%		
16	OE = 50%		OE = 25%		MT^12e2^	NM	NM	OE = 65%	OE = 0%	OE = 0%		OE = 0%		OE = 0%		OE = 10%		
17	OE = 30%		OE = 20%		NM	NM	NM	OE = 60%	OE = 0%	OE = 0%		OE = 0%		OE = 0%		OE = 5%		
18	OE = 25%		OE = 25%		NM	NM	NM	OE = 30%	OE = 0%	OE = 0%		OE = 0%		OE = 0%		OE = 10%		
19	OE = 20%		OE = 30%		MT^12e2^	NM	NM	OE = 65%	OE = 0%	OE = 0%		OE = 0%		OE = 0%		OE = 10%		
20	OE = 25%		OE = 35%		NM	MT^61e3^	NM	OE = 70%	OE = 10%	OE = 0%		OE = 0%		OE = 0%		OE = 10%		
21	OE = 80%		OE = 60%		NM	NM	NM	OE = 61%	OE = 0%	OE = 0%		OE = 45%		OE = 0%		OE = 10%		Cetuximab (250 mg/m^2^)
22	OE = 65%		OE = 80%		MT^12e2^	NM	NM	OE = 83%	OE = 0%	OE = 0%		OE = 0%		OE = 0%		OE = 10%		Bevacizumab (5 mg/kg)
23	OE = 30%		OE = 30%		NM	NM	NM	OE = 50%	OE = 0%	OE = 0%		OE = 0%		OE = 0%		OE = 5%		
24	OE = 45%		OE = 45%		NM	NM	NM	OE = 80%	OE = 0%	OE = 0%		OE = 0%		OE = 0%		OE = 10%		
25	OE = 50%		OE = 80%		NM	NM	NM	OE = 60%	OE = 0%	OE = 0%		OE = 0%		OE = 0%		OE = 8%		Bevacizumab (5 mg/kg)
26	OE = 60%		OE = 90%		NM	NM	NM	OE = 70%	OE = 0%	OE = 0%		OE = 0%		OE = 0%		OE = 10%		Bevacizumab (5 mg/kg)
27	OE = 20%		OE = 45%		NM	NM	NM	OE = 58%	OE = 0%	OE = 0%		OE = 0%		OE = 0%		OE = 10%		
28	OE = 20%		OE = 45%		MT^12e2^	NM	NM	OE = 60%	OE = 0%	OE = 0%		OE = 0%		OE = 0%		OE = 10%		
29	OE = 60%		OE = 40%		NM	NM	NM	OE = 78%	OE = 0%	OE = 0%		OE = 45%		OE = 0%		OE = 10%		Cetuximab (250 mg/m^2^)
30	OE = 20%		OE = 45%		MT^12e2^	NM	NM	OE = 62%	OE = 0%	OE = 0%		OE = 0%		OE = 0%		OE = 10%		
31	OE = 45%		OE = 30%		NM	NM	NM	OE = 65%	OE = 10%	OE = 0%		OE = 0%		OE = 0%		OE = 10%		
32	OE = 60%		OE = 55%		NM	NM	NM	OE = 60%	OE = 0%	OE = 0%		OE = 0%		OE = 0%		OE = 10%		Cetuximab (250 mg/m^2^)
33	OE = 10%		OE = 45%		NM	NM	NM	OE = 42%	OE = 0%	OE = 0%		OE = 0%		OE = 0%		OE = 10%		
34	OE = 20%		OE = 20%		NM	NM	NM	OE = 70%	OE = 10%	OE = 0%		OE = 0%		OE = 0%		OE = 10%		
35	OE = 70%		OE = 55%		NM	NM	NM	OE = 58%	OE = 0%	OE = 0%		OE = 0%		OE = 0%		OE = 12%		Cetuximab (250 mg/m^2^)
36	OE = 40%		OE = 45%		NM	NM	NM	OE = 70%	OE = 0%	OE = 0%		OE = 0%		OE = 0%		OE = 10%		
37	OE = 50%		OE = 50%		NM	MT^61e3^	NM	OE = 65%	OE = 0%	OE = 0%		OE = 0%		OE = 5%		OE = 25%		
38	OE = 20%		OE = 30%		NM	NM	NM	OE = 70%	OE = 5%	OE = 0%		OE = 0%		OE = 0%		OE = 10%		
39	OE = 20%		OE = 20%		MT^12e2^	NM	NM	OE = 75%	OE = 15%	OE = 0%		OE = 0%		OE = 0%		OE = 25%		
40	OE = 30%		OE = 30%		NM	NM	NM	OE = 80%	OE = 0%	OE = 0%		OE = 0%		OE = 0%		OE = 10%		
41	OE = 10%		OE = 45%		NM	NM	NM	OE = 65%	OE = 0%	OE = 0%		OE = 0%		OE = 0%		OE = 20%		
42	OE = 50%		OE = 80%		MT^12e2^	NM	NM	OE = 70%	OE = 0%	OE = 0%		OE = 0%		OE = 0%		OE = 10%		Bevacizumab (5 mg/kg)
43	OE = 60%		OE = 30%		NM	NM	NM	OE = 70%	OE = 0%	OE = 0%		OE = 0%		OE = 0%		OE = 10%		Cetuximab (250 mg/m^2^)
44	OE = 45%		OE = 55%		MT^12e2^	NM	NM	OE = 70%	OE = 0%	OE = 0%		OE = 0%		OE = 0%		OE = 10%		Bevacizumab (5 mg/kg)

*Pt* patient, *IV-CTCs* isolated viable circulating tumor cells, *5-FU* 5 fluorouracil, *5-FU* 5 fluorouracil, *MMC* mitomycin, *IRI* irinotecan, *OX* oxaliplatin, *TC* tailored chemotherapy, *CST* chemosensitivity tests, *S* sensitivity, *OE* gene over-expression, *OE* 0% includes down-regulated gene expression, *NM* no mutations, *MT* mutated type, *12e2* codon 12 of exon 2, *61e3* codon 61 of exon 3, *EGFR* epidermal growth factor receptor, *VEGFR* vascular endothelial growth factor receptor, *KRAS* Kirsten rat sarcoma virus, *NRAS* neuroblastoma RAS viral oncogene homolog, *BRAF* v-Raf murine sarcoma viral oncogene homolog B gene, *MDR1* Multidrug resistance gene (ABCB1 gene), *TYMS* thymidylate synthase gene, *DHFR* dihydrofolate reductase, *ERCC1* DNA excision repair protein, *GST* glutathione S-transferases

In the HAI/target therapy cohort, HAI plus chemo-filtration was repeated for a maximum of three times at 6/8-week intervals, with cetuximab or bevacizumab administered every week up to disease progression or unacceptable toxicity. In both cohorts, treatment was discontinued upon disease progression (i.e., > 20% increase in CRCLM or distant relapse dimension and/or detection of simultaneous additional relapse); a worsening of general condition (Child–Pugh C liver function and/or ECOG performance status of > 3); severe adverse events or patient withdrawal.

### Response assessment

Enhanced computed tomography (CT) or magnetic resonance imaging (MRI) was reviewed for each patient 6–8 weeks following the first cycle of third-line therapy and objective response (ORR) and disease control (DCR) rates were evaluated 5/6 months following the first cycle of third-line therapy, using Response Evaluation Criteria in Solid Tumor (RECIST) software (version 1.1). The responses of patients treated prior to 2009 were re-classified retrospectively. Adverse reactions were evaluated using National Cancer Institute-Common Terminology Criteria for Adverse Events software (version 4.03) and classified from 0–4. Quality of life (QoL) was also evaluated 5/6 months following the first cycle of third-line therapy and graded according to the Eastern Cooperative Oncology Group (ECOG) criteria. The last follow-up date in this study was November 15th, 2019.

### Statistical analysis

Statistical analyses were performed using STATA software (version 14, Stata Corp, College Station, TX). Continuous variables are displayed as the mean ± standard deviation (SD) and median with interquartile range (iqr), and qualitative data are expressed as frequency or percentage. Student *t*, Chi-square, or Fisher’s exact tests were applied to numerical data and Kaplan–Meier tests were used to estimate OS and PFS survival rates, with significant differences determined by log-rank test.

Clinical prognostic variables included age, gender, tumor primary site and pathological grade, time to developing liver metastasis, bilateral liver involvement, ≥ 1 metastasis of > 5 cm diameter, extra-hepatic metastasis, ECOG grading, and KRAS, NRAS, and BRAF mutational status. Univariate analysis was applied to all variables, with variables of *P *value < 0.10 entered into multivariate analysis. Cox-model prognostic factor identification was related to survival and statistical significances associated with a probability (*P*) ≤ 0.05.

## Results

### Tumor response in the two cohorts

The 44 patient HAI/target therapy cohort was characterised by: 1/44 (2.27%) complete responses (CRs); 17/44 (38.63%) partial responses (PRs); 25/44 (56.81%) stable diseases (SDs), and 1/44 (2.27%) progressive disease (PDs), and the 62 patient systemic therapy cohort characterised by: 1/62 (1.6%) CR; 11/62 (17.74%) PRs; 23/62 (37.09%) SDs, and 28/62 (45.16%) PDs. Both ORR and DCR values in the HAI/target therapy cohort were significantly higher (P < 0.014, Fisher’s exact test) than in the systemic therapy cohort (40.91 and 97.73% verses 17.74 and 54.84%, respectively).

### Adverse events

No technical or vascular complications were detected during infusion in the HAI/target therapy cohort and there were no infusion-related postoperative deaths. However, one patient (2.27%) developed an inguinal haematoma. Comparative evaluation (Table 3) detected significantly higher rates of grade 1–2 nausea/vomiting, diarrhea, anorexia, fatigue, mucositis, hand foot syndrome, rash, and arterial hypertension in the systemic therapy cohort. Non-infectious fever was the only parameter that was significantly higher in the HAI/target therapy cohort.

**Table 3:** Adverse events in the two CRCLM patient cohorts after the first cycle of the third-line treatment

Adverse events	HAI/target therapy cohort (*n* = 44)			Systemic therapy cohort (*n* = 62)			*χ*^2^	*P* value
	Grade	*n*	%	Grade	*n*	%		
Bone marrow hypocellularity	− 1/2	6	13.64	− 1/2	5	8.06	0.86	0.35
	− 3/4	1	2.27	− 3/4	8	12.90	3.74	0.06
Liver function								
AST and ALT increased	− 1/2	3	6.82	− 1/2	6	9.68	0.27	0.60
	− 3/4	1	2.27	− 3/4	3	4.83	0.47	0.49
Total bilirubin increased	− 1/2	2	4.55	− 1/2	5	8.06	0.52	0.47
	− 3/4	0	0	− 3/4	1	1.61	n.a	
Ascites/itch	− 1/2	0	0	− 1/2	3	4.83	n.a	
	− 3/4	0	0	− 3/4	0	0	n.a	
Gastrointestinal symptoms								
Nausea/vomiting	− 1/2	7	15.90	− 1/2	29	46.77	10.93	0.001
	− 3/4	3	6.82	− 3/4	1	1.61	1.92	0.17
Diarrhea	− 1/2	3	6.82	− 1/2	41	66.13	37.29	0.001
	− 3/4	0	0	− 3/4	4	6.45	n.a	
Constipation	− 1/2	0	0	− 1/2	4	6.45	n.a	
	− 3/4	0	0	− 3/4	0	0	n.a	
Anorexia	− 1/2	0	0	− 1/2	16	25.80	13.37	0.001
	− 3/4	0	0	− 3/4	0	0	n.a	
Dysgeusia	− 1/2	0	0	− 1/2	2	3.28	n.a	
	− 3/4	0	0	− 3/4	0	0	n.a	
Other								
Abdominal pain	− 1/2	2	4.55	− 1/2	1	1.61	0.80	0.37
	− 3/4	0	0	− 3/4	2	3.23	n.a	
Non-infectious fever	− 1/2	18	40.90	− 1/2	3	4.83	21.08	0.001
	− 3/4	2	4.55	− 3/4	1	1.61	0.80	0.37
Multiple hepatic abscesses	− 1/2	0	0	− 1/2	0	0	n.a	
	− 3/4	1	2.27	− 3/4	0	0	n.a	
Neuropathy	− 1/2	1	2.27	− 1/2	4	6.45	1.00	0.32
	− 3/4	0	0	− 3/4	1	1.61	n.a	
Oxaliplatin allergy	− 1/2	1	2.27	− 1/2	0	0	n.a	
	− 3/4	0	0	− 3/4	1	1.61	n.a	
Alopecia	− 1/2	1	2.27	− 1/2	2	3.23	0.08	0.77
	− 3/4	3	6.82	− 3/4	0	0	n.a	
Fatigue	− 1/2	3	6.82	− 1/2	39	62.90	33.84	0.001
	− 3/4	0	0	− 3/4	2	3.23	n.a	
Mucositis	− 1/2	5	0	− 1/2	27	43.54	12.65	0.001
	− 3/4	0	0	− 3/4	2	3.23	n.a	
Hand–Foot Syndrome	− 1/2	0	0	− 1/2	11	17.74	8.71	0.003
	− 3/4	0	0	− 3/4	0	0	n.a	
Rash	− 1/2	5	0	− 1/2	8	12.90	0.06	0.81
	− 3/4	0	0	− 3/4	2	3.23	n.a	
Rhinitis/epistaxis	− 1/2	7	15.90	− 1/2	16	25.80	1.48	0.22
	− 3/4	0	0	− 3/4	0	0	n.a	
Paronychia	− 1/2	0	0	− 1/2	5	8.06	3.72	0.06
	− 3/4	0	0	− 3/4	0	0	n.a	
Deep vein thrombosis	− 1/2	0	0	− 1/2	2	3.23	n.a	
	− 3/4	0	0	− 3/4	0	0	n.a	
Conjunctivitis	− 1/2	0	0	− 1/2	5	8.06	3.72	0.06
	− 3/4	0	0	− 3/4	0	0	n.a	
Kidney failure	− 1/2	0	0	− 1/2	2	3.23	n.a	
	− 3/4	0	0	− 3/4	2	3.23	n.a	
Arterial hypertension	− 1/2	0	0	− 1/2	14	22.58	11.45	0.001
	− 3/4	0	0	− 3/4	0	0	n.a	
Dysphonia	− 1/2	0	0	− 1/2	5	8.06	3.72	0.06
	− 3/4	0	0	− 3/4	0	0	n.a	

*n.a.* not applicable

### Quality of life in the two cohorts

ECOG-score quality of life was measured prior to and 3 months, following the first cycle of third-line treatment regimens. ECOG 1-to-ECOG 2 worsening was observed in 4.84% of the systemic therapy cohort. In contrast, 22.73% of the HAI/target therapy cohort exhibited ECOG 2-to-ECOG 1 improvement.

### Patient cohort survival times

In 106 patients with unresectable CRCLM, median survival time from diagnosis was 40 months (iqr 30–52), and median survival time from unresectable CRCLM diagnosis to death or last contact was 37.5 months in the HAI/target therapy cohort and 32 months in the systemic therapy cohort, but was not statistically significant (*P* = 0.91).

**Fig. 2:**
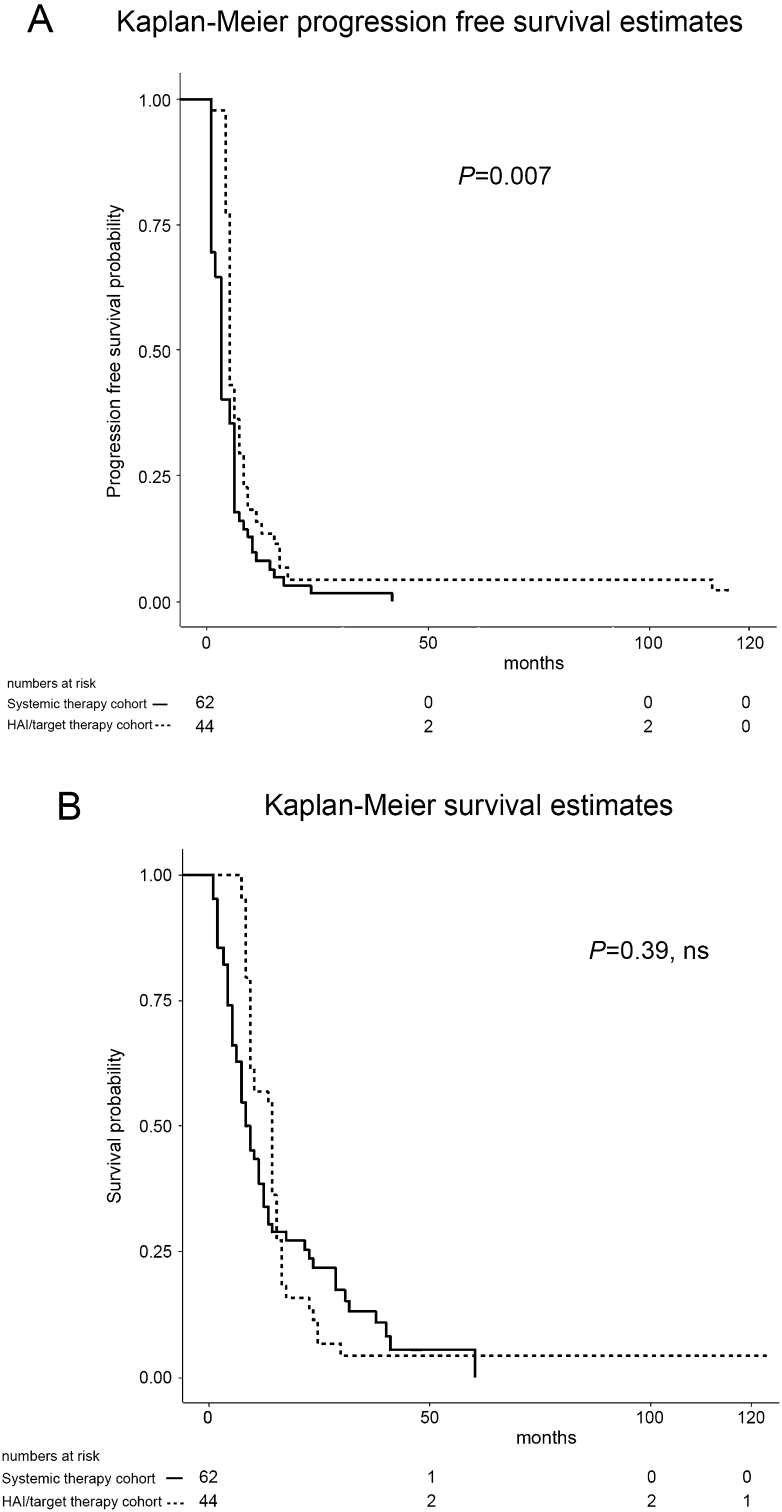
Kaplan–Meier progression-free (**a**) and overall (**b**) survival estimates for 106 patients with CRCLM from the first cycle of third-line therapy to time-of-death or end of follow-up

Median progression-free survival (PFS), from the first cycle of third-line therapy to death or last contact, was 5 months in the HAI/target therapy cohort, which was significantly longer than 3 months in the systemic therapy cohort (*P* < 0.007, Fig. 2a). Univariate PFS prognostic factor analysis is reported in Table 4 (Part A) and no significant prognostic factors were identified by multivariate analysis in the HAI/target therapy cohort. In contrast, bilateral liver involvement (HR = 1.75, 95% CI = 0.99–3.09) and EGOG grade 2 (HR = 2.57, 95% CI = 1.17–5.64) were both identified as significant PFS prognostic factors in the systemic therapy cohort.

**Table 4:** Univariate analysis for prognostic factors; Part A: median progression-free survival (PFS) times and Part B: median survival times, from first cycle of third-line therapy

Part A						
Variables	HAI/target therapy cohort (*n* = 44)			Systemic therapy cohort (*n* = 62)		
	PFS (months) [n.pts]	Log-rank *χ*^2^ (*P* value)	Cox HR, (95%CI)	PFS (months) [n.pts]	Log-rank *χ*2 (*P* value)	Cox HR, (95%CI)
Age						
< 66 years	5 [*n* = 21]	0.50		3 [*n* = 41]	0.01	
- ≥ 66 years	5 [*n* = 23]	(0.48)		3 [*n* = 21]	(0.93)	
Gender						
Females	6 [*n* = 19]	1.90		3 [*n* = 23]	0.60	
Males	5 [*n* = 25]	(0.17)		3 [*n* = 39]	(0.44)	
Primary tumor site						
Right hemicolon	5 [*n* = 10]			3 [*n* = 17]		
Left hemicolon	5 [*n* = 27]	0.60		2.5 [*n* = 26]	1.14	
Rectum	5 [*n* = 7]	(0.74)		3 [*n* = 19]	(0.56)	
Primary tumor pathological grade						
Well or intermediate	5 [*n* = 23]	3.14		3 [*n* = 31]	0.05	
Poor	5 [*n* = 21]	(0.08)		3 [*n* = 31]	(0.83)	
Time to liver metastases						
Synchronous	5 [*n* = 27]	0.10		3 [*n* = 48]	0.03	
Metachronous	5 [*n* = 17]	(0.75)		3 [*n* = 14]	(0.87)	
Bilateral liver involvement						
Yes	5.5 [*n* = 22]	0.31		3 [*n* = 42]	5.97	1.78
Not	5 [*n* = 22]	(0.57)		6 [*n* = 20]	(0.01)	(1.02–3.09)
Presence of at least one metastasis with diameter > 5 cm						
Yes	5 [*n* = 19]	5.97	1.94	3 [*n* = 31]	1.13	
Not	6 [*n* = 25]	(0.01)	(1.04–3.64)	3 [*n* = 31]	(0.29)	
Presence of extra-hepatic metastasis						
Yes	5 [*n* = 22]	3.52		3 [*n* = 43]	6.29	1.87
Not	6.5 [*n* = 22]	(0.06)		5 [*n* = 19]	(0.01)	(1.04–3.37)
ECOG classification						
0				3 [*n* = 34]		
1	6.5 [*n* = 24]	5.21	1.86	5 [*n* = 19]	6.85	2.27
2	5 [*n* = 20]	(0.02)	(0.99–3.46)	2 [*n* = 9]	(0.03)	(1.04–4.98)
KRAS, NRAS, and BRAF status						
No mutations	5.5 [*n* = 30]	2.25		3 [*n* = 38]	0.49	
Mutations	5 [*n* = 14]	(0.13)		3 [*n* = 24]	(0.48)	

Part B						
Variables	HAI/target therapy cohort (*n* = 44)			Systemic therapy cohort (*n* = 62)		
	Survival/3rd line (months) [n.pts]	Log-rank *χ*^2^ (*P* value)	Cox HR, (95%CI)	Survival/3rd line (months) [n.pts]	Log-Rank *χ*^2^ (*P* value)	Cox HR, (95%CI)
Age						
< 66 years	9 [*n* = 21]	2.17		8 [*n* = 41]	0.28	
≥ 66 years	14 [*n* = 23]	(0.14)		9 [*n* = 21]	(0.60)	
Gender						
Female	14 [*n* = 19]	1.25		8 [*n* = 23]	0.02	
Male	10 [*n* = 25]	(0.26)		9 [*n* = 39]	(0.88)	
Primary tumor site						
Right hemicolon	11 [*n* = 10]			11 [*n* = 17]		
Left hemicolon	14 [*n* = 27]	4.12		6.5 [*n* = 26]	1.84	
Rectum	15 [*n* = 7]	(0.13)		9 [*n* = 19]	(0.40)	
Primary tumor pathological grade						
Well or intermediate	15 [*n* = 23]	5.50	1.96	10 [*n* = 31]	0.16	
poor	9 [*n* = 21]	(0.02)	(1.04–3.69)	8 [*n* = 31]	(0.69)	
Time to liver metastases						
Synchronous	9.5 [*n* = 27]	0.21		8.5 [*n* = 48]	0.16	
Metachronous	14 [*n* = 17]	(0.65)		9 [*n* = 14]	(0.69)	
Bilateral liver involvement						
Yes	13.5 [*n* = 22]	0.01		7 [*n* = 42]	6.41	2.06
Not	14 [*n* = 22]	(0.92)		15.5 [*n* = 20]	(0.01)	(1.14–3.70)
Presence of at least one metastasis with diameter > 5 cm						
Yes	9 [*n* = 19]	10.08	2.46	6 [*n* = 31]	5.61	1.86
Not	15 [*n* = 25]	(0.001)	(1.31–4.63)	13 [*n* = 31]	(0.02)	(1.09–3.20)
Presence of extra-hepatic metastasis						
Yes	9 [*n* = 22]	1.89		7 [*n* = 43]	5.63	2.02
Not	14 [*n* = 22]	(0.17)		12 [*n* = 19]	(0.02)	(1.10–3.72)
ECOG classification						
0				10 [*n* = 34]		
1	15 [*n* = 24]	11.4	2.59	11 [*n* = 19]	8.38	1.47
2	9 [*n* = 20]	(0.001)	(1.38–4.88)	5 [*n* = 9]	(0.01)	(0.99–2.20)
KRAS, NRAS, and BRAF genotype status						
No mutations	14 [*n* = 30]	1.72		11 [*n* = 38]	2.15	
Mutations	9 [*n* = 14]	(0.19)		7 [*n* = 24]	(0.14)	

*HR* hazard ratio, *CI* confidence interval, *ns* not significant, *Pts* patients

In the HAI/target therapy cohort, the median survival time from the first cycle of third-line therapy to death or last contact of 14 months was longer than 8.5 months in the systemic therapy cohort, but this was not statistically significant (*P* = 0.39, Fig. 2b). Univariate survival prognostic factor analysis, following third-line therapy, is reported in Table 4 (Part B). Multivariate analysis identified ≥ one metastasis of diameter > 5 cm (HR = 2.05, 95% CI = 1.01–4.16) and EGOG grade 2 (HR = 2.27, 95% CI = 1.16–4.43) as significant survival prognostic factors in the HAI/target therapy cohort, and identified bilateral liver involvement (HR = 2.31, 95% CI = 1.18–4.52), extra-hepatic metastases (HR = 1.98, 95% CI = 1.06–3.69), and EGOG grade 2 (HR = 3.42, 95% CI = 1.43–8.21) as significant prognostic factors in the systemic therapy cohort.

## Discussion

We report that HAI with chemo-filtration, using drug regimens and subsequent target therapy selected by liquid biopsy precision oncotherapy, included in the multidisciplinary treatment of unresectable CRCLM, progressing after at least two lines of systemic therapy, is both safe and tolerable. In the cohort of CRCLM patients studied, the procedure was simple to perform, repeatable, associated with minimal systemic cytotoxicity, resulted in an ORR of ≈ 40%, DCR of ≈ 97%, median PFS of 5 months and median OS of 14 months, from the first HAI cycle to time-of-death or last follow-up and in the real-life setting may, therefore, have significant advantages over current third-line systemic therapy in terms of local disease control, PFS, and ECOG performance status, with ECOG grade 2 identified as the most unfavourable prognostic factor in both treatment strategies.

With respect to CTCs purification methodology, blood preservation and transport were in line with the previous reports (Qin et al. 2014; Kang et al. 2016), CTCs’ purification and characterization in line with recent approved clinical and regulatory improvements liquid biopsy use in diagnostics (Goodsaid 2019) and PCR-based techniques for precision oncotherapy in accordance with the recent guidelines for colorectal cancer treatment ( Kentaro et al. 2018).

Consistent with the great potential of liquid biopsy precision oncotherapy to improve therapeutic strategies and bolster clinical and patient expectations (Karachaliou et al. 2015; Goodsaid 2019), chemosensitivity tests performed on CTCs from patients previously treated with systemic chemotherapy were not only useful in identifying potentially efficacious drug regimens for HAI, but also provided important CTC gene expression profile information of potential relevance to drug resistance and future target-therapy efficacy.

Considering that mitomycin is not recommended for colorectal cancer (Fiorentini et al. 2017a), it was intriguing that CTCs from 47% of patients exhibited high sensitivity to mitomycin, compared to irinotecan (27%), oxaliplatin (22%), and 5-fluorouracil (only 2%). This may be explained by the previous systemic chemotherapy for metastatic disease and/or high over-expression (≥ 70%) of the multi-drug resistance gene MDR1 in CTCs from 41% of patients. Analysis of CTCs tumor gene expression also revealed over-expression of EGFR and/or VEGFR in 51% of patients, not previously submitted to target therapy, of which 30% (10 patients) were successively treated with third-line targeted therapy.

Compared to our previous reports concerning untreated patients or patients pre-treated with single-line therapy, this current study concerning patients in progression after at least two lines of systemic therapy, resulted in a median OS of 14 months, which represents a significant improvement. A previous prospective multicentre phase II trial (OPTILIV) (Lévi et al. 2016) reported a median OS of 18.7 months (PFS of 8.6 months) for patients with unresectable CRCLM in progression after 1–3 systemic therapy regimens, submitted for HAI, via fully implanted catheters and access ports, with irinotecan (180 mg/m^2^), oxaliplatin (85 mg/m^2^) and 5-fluorouracil (2800 mg/m^2^), followed by i.v. cetuximab (500 mg/m^2^). In the OPTILIV study, 17% of patients did not receive therapy for problems associated with permanent catheter implantation, HAI was interrupted in 67% of patients for catheter-related complications and 77% of patients suffered grade 3–4 toxicity (mainly neutropenia, abdominal pain, fatigue, and diarrhea). In the present study, no procedure-related complications were associated with temporary catheterization and the addition of chemo-filtration reduced grade 3 haematological toxicity to < 3% and abnormal liver function to < less 15% of patients. Multiple hepatic abscesses, however, developed in one patient.

With respect to third-line locoregional therapy for patients with unresectable CRCLM, the present study can be compared to two studies of raltitrexed-based TACE (Guo et al. 2017; Wei et al. 2019). In the Fudan University Shanghai Cancer Center/Xintai Taishan Medical University study (Wei et al. 2019), a median OS of 14 months (PFS of 2.1 months) was reported for raltitrexed (4 mg) combined with oxaliplatin (100 mg) and pirarubicin (60 mg), whereas a median OS of 20.6 (PFS of 4.9 months) was reported in the Peking University Cancer Hospital study (Guo et al. 2017) for epirubicin (40 mg plus spongostan particles and iodized oil/lipiodol), followed by 48 h HAI with raltitrexed (3 mg/m^2^) combined with oxaliplatin (85 mg/m^2^) and 5-fluorouracil (2000 mg/m^2^). The small-sample size (18 patients) in the latter study and failure to report prior first- and second-line systemic chemotherapeutic regimens in both studies, however, limit further discussion of the impressive 20.6-month OS in Peking University Cancer Hospital study (Guo et al. 2017). The current study, however, should not be compared to our previous TACE studies, which were performed without chemo-filtration on patients with and without unresected primary tumours, in progression after a single line of systemic therapy (Fiorentini et al. 2017b,^2018^,^2020^).

In spite of limitations including: a relatively small-sample sizes; differences in previous systemic chemotherapy regimens; differences in ECOG performance status; potential selection bias based on the percentage of additional metastatic sites; potential treatment bias based on non-homogeneous precision oncotherapy-selected drug regimens; and potential control group selection bias based on inclusion of patients treated with systemic therapy without locoregional chemotherapy, we propose the use of HAI with drug regimens selected by liquid biopsy precision oncotherapy in a multidisciplinary strategy for the treatment of unresectable CRCLM in progression after two lines of systemic therapy. Furthermore, we suggest that HAI could be extended beyond three courses, repeatable liquid biopsies could also be used to select systemic therapeutic regimens and to expand testing to the other drugs (Cappabianca et al. 2019; Morelli et al. 2010; Bruera et al. 2012). Therefore, we stress the need for a future multicentre prospective phase III study to fully confirm the efficacy of HAI with liquid biopsy-selected drug regimes for the treatment of unresectable, recurrent, and refractory CRCLM.
